# Atopy, passive smoking, respiratory infections and asthma among children from kindergarten and elementary school

**DOI:** 10.1590/S1516-31802002000400004

**Published:** 2002-07-07

**Authors:** Sandra Aparecida Ribeiro, Tatiana Furuyama, Simone Schenkman, José Roberto de Brito Jardim

**Keywords:** Respiratory infections, Asthma, Children, Passive smoking, Allergy, Infecções respiratórias, Asma, Crianças, Fumo passivo, Alergia

## Abstract

**CONTEXT:**

It has been demonstrated that children exposed to parents who smoke have more respiratory infections and asthma.

**OBJECTIVE:**

To study the association of both respiratory infections and asthma attacks with atopy, passive smoking and time spent daily at school, among children aged 4 to 9 years old from a kindergarten and elementary school in the city of São Paulo between May and July of 1996.

**TYPE OF STUDY:**

Descriptive study.

**SETTING:**

A kindergarten and elementary school with linkages to Universidade Federal de São Paulo/ Escola Paulista de Medicina.

**PARTICIPANTS:**

183 children between 4 and 9 years old.

**MAIN MEASUREMENTS:**

A questionnaire consisting of 31 questions was answered by the parents of 183 children, and skin tests for inhaled antigens were performed on 88 children whose parents had given prior agreement for the procedure.

**RESULTS:**

Among the children, 51% had had respiratory infections during the preceding 3 months and 25.7% were asthmatic, of whom 52.1% had had one or more asthma attacks during the preceding 3 months. Children exposed to passive smoking did not have more respiratory infections or asthma attacks in comparison with those not exposed. We observed a significant association between atopic disorders in parents and children who were not exposed to passive smoking. There were also associations between atopic disorders in parents and asthma attacks in their infants, and between such disorders and a higher incidence of respiratory infections in the infants during the preceding 3 months. However, the presence of two or more positive skin tests for allergies did not have a correlation with respiratory infections and asthma attacks in this sample. In addition to this, children who studied full time at school did not have a higher occurrence of respiratory infections and asthma attacks.

**CONCLUSIONS:**

The presence of respiratory infections and asthma was associated with atopic parents but not with the presence of two or more positive skin tests for allergies among the children. Also, respiratory infections and asthma attacks were not associated with smoking parents or with the length of time spent by the children at school.

## RESUMO

**CONTEXTO:**

Foi demonstrado que crianças de pais fumantes estão mais predispostas a infecções respiratórias e asma.

**OBJETIVO:**

Estudar a associação de infecções respiratórias e crises de asma em crianças de quatro a nove anos de idade, matriculadas em uma escola que funciona como pré-escola e 1^o.^ grau em São Paulo, Capital, nos meses de maio a julho de 1996, com presença de atopia, exposição ao fumo passivo e tempo de permanência na escola (parcial ou integral).

**TIPO DE ESTUDO:**

Estudo descritivo.

**LOCAL:**

Pré-escola e de primeiro grau da Universidade Federal de São Paulo.

**CASUÍSTICA:**

183 crianças, sendo 104 meninos e 79 meninas, entre 4 e 9 anos.

**VARIÁVEIS ESTUDADAS:**

Foi administrado um questionário contendo 31 questões aos responsáveis pelas crianças e realizados 88 testes cutâneos de alergia para inalantes, nas crianças cujos pais haviam fornecido consentimento prévio.

**RESULTADOS:**

Do total de crianças, 51% tiveram infecções respiratórias nos últimos três meses e 25,7% eram asmáticas, sendo que 52,1% delas tiveram uma ou mais crises nos últimos três meses. As crianças expostas à fumaça de cigarro no domicílio não tiveram mais infecções respiratórias e crises de asma que as não-expostas. A positividade a dois ou mais testes cutâneos de alergia não se correlacionou significantemente à presença de infecções respiratórias e crises de asma nas crianças estudadas, embora tenha havido associação estatisticamente significante entre pais com história de atopia e filhos com mais infecções respiratórias e/ou crises de asma, assim como pais com história de atopia e filhos com menor exposição à fumaça de cigarro. As crianças com permanência em tempo integral na escola não ti-veram maior ocorrência de infecções respiratórias e crises de asma.

**CONCLUSÃO:**

A presença de infecções respiratórias e crises de asma está associada à atopia dos pais, mas não à presença de positividade a dois ou mais testes cutâneos de alergia nas crianças. Por outro lado, a presença de infecções respiratórias e crises de asma não estiveram associadas com exposição passiva à fumaça de cigarro no domicílio e com tempo de permanência na escola.

## INTRODUCTION

Smoking is a major public health issue due to its direct and indirect effects on health outcomes.^1,2,3^ It has been demonstrated that exposure to passive smoking (tobacco smoke) increases the incidence of coughing and otitis, as well as generating childhood asthma and bronchial hyper-responsiveness, acute bronchiolitis, lower respiratory tract infections and pneumonia.^1,4,5,6^ The number of smokers in the home and the quantity of cigarettes consumed has been correlated with the abovementioned health problems. In addition, a linear correlation has been shown between smoking mothers and respiratory disease in children.^2,7^

Passive smoking may affect children directly, by decreasing pulmonary function, or indirectly^8,9^ by increasing their exposure to infectious diseases, since smokers have a higher incidence of respiratory infections.^1,2,3^

Three cohort studies have demonstrated an increase in the frequency of respiratory diseases and hospital admissions among toddlers whose parents smoke. In Israel, a survey of 10,762 children aged less than one year old revealed 9.5 admissions due to bronchitis and pneumonia per 100 children whose mothers were non-smokers, in comparison with 13.1 admissions per 100 children with smoking mothers.

British researchers conducted a follow-up study on 2,205 children for 5 years and showed that the incidence of lower respiratory tract infection was increased in children of smoking mothers in comparison with children of non-smoking mothers. This incidence increased considerably when both parents smoked, even after adjusting for the number of members in the family, socioeconomic status and birth weight.^2^

A third cohort study conducted in New Zealand, with 1,265 children followed up for 3 years, exhibited the following results: maternal smoking was strongly related to bronchitis/pneumonia in the first year of life, and an increase of 5 cigarettes per day resulted in an increment of 2.5 to 3.5 cases of bronchitis/pneumonia for every 100 children at risk.^3^

The presence of smoking adults in the family increases the lengths of time spent in bed and activity restrictions among children. Disease duration (in days) increased according to the number of cigarettes smoked in the family.^1^

The aim of this work was to study the association between parents’ smoking habits and the incidence of respiratory infections and asthma attacks during the preceding 3 months, among children who were attending a kindergarten and elementary school with linkages to an university, in relation to their socioeconomic status, positive response rate to skin tests for allergies, breastfeeding history, atopic disorders among their parents, presence of pets at home and time spent at school.

## METHODS

### Design.

Descriptive study.

### Setting.

The study was undertaken at the kindergarten and elementary school of Universidade Federal de São Paulo, a public institution.

### Participants.

183 children aged 4 to 9 years old, during the months of May through July 1996.

### Procedures and main measurements.

The parents (or whoever was responsible for the children) filled out a standardized questionnaire consisting of 31 items that inquired about smoking characteristics (such as who smokes, how many people smoke, how many cigarettes per day) and also ex-smokers’ characteristics (why they quit the habit). The other covariates considered were the presence of siblings; their ages; whether they were breastfed or not; the frequency of respiratory infections during the last 3 months; the presence of asthma/bronchitis and its characteristics (how many attacks, age of onset of disease and regularity or treatment); and any history of atopic disorders in parents. In the last part, a socioeconomic evaluation was attempted, verifying housing conditions, number of rooms and number of people living in the household, head of family, educational level, family income and number of housing utilities, according to the criteria.^10^

Skin tests for allergies were performed on 88 children, following these steps: 1) sterilization of the forearm using alcohol; 2) scarification using a disposable thin blade, at 8 different places in the forearm; 3) application of 1 drop of allergen (home dust with Dermatophagoides, tobacco, cotton, fungi, dog hair and epithelium), a positive control (histamine) and a negative control (saline); 4) after 20 minutes, the reading was performed: (-) negative test: no papule or erythema in comparison with the negative control, (-/+) dubious test: papule less than 5 mm with moderate erythema, (+) weakly positive: papule with a diameter of 5 mm and moderate erythema, (++) mildly positive test: papule 5-10 mm, without pseudopodia and with moderate to strong erythema, (+++) strongly positive papule of 10 mm or more, with pseudopodia and significant erythema. Tests were considered positive when the readings were weakly, moderately or strongly positive. For the allergy tests, we excluded children under 4 years old and children whose parents had not given permission for the tests to be performed.

This study was approved by the Medical Ethics Committee of UNIFESP (Universidade Federal de São Paulo).

#### Statistical methods.

Pearson Chi-Squared and Fisher exact tests were performed (when at least one expected frequency was less than 5). Values of p < 0.05 were considered as significant. The odds ratio and 95% confidence interval were calculated to evaluate the presence of associations between respiratory morbidity in the children and environmental variables. Data were analyzed using the Stata statistical package.

## RESULTS

We sent out 255 questionnaires, and 183 responses were returned by the parents or whoever was responsible for the children in the sample (a return rate of 71.8%). In 37.8% of the homes, the father and/or mother were smokers and in 47.2% there was another smoker in the house. Only in 15% of the houses were there no smokers.

Table 1 shows the different kinds of dwellings occupied by the families, in relation to occupation conditions, number of rooms, number of people living together and ventilation conditions. Most children shared their room with someone else (88.7%), and about 40% of them had pet animals at home, half of which were reported to be dogs.

**Table 1:** Types of housing, housing conditions, number of rooms, number of people living there and ventilation

Type	Occupation	No. of rooms	No. of people living there	Ventilation
House (65.0%)	own (55.9%)	2 to 4 (44.7%)	2 to 4 (66.1%)	good ventilation (36.8%)
Apartment (33.9%)	rented (26.3%)	5 or more (55.3%)	5 or more (33.9%)	sunny (35.2%)
Other (1.1%)	other (17.8%)			musty (28.0%)

In relation to the socioeconomic status of the children, according to the ABA/ABIPEME criteria, it was verified that most families were located in the B (35.2%) and C (50.6%) classes.

There was a history of breastfeeding in 92.3% of the responses, and 50% maintained it for at least the first four months.

With regard to the length of time spent at school each day, 76.1% of the children attended full-time, while only 23.8% attended part-time. Children who studied full-time were equally exposed to passive smoking at home, when compared with children that studied part-time. We did not find any statistical difference for the incidence of respiratory infections and asthma in relation to the length of time spent at school.

Eighty-eight skin tests for allergies (on 48.1% of the sample, of whom 57% were boys and 43% were girls) were performed with immediate reading for 6 inhaled allergens. From these tests, 53.4% did not exhibit a positive response to the tested antigens. The percentage of positive responses to the antigens, separately, were 29.5% for dog epithelium, 21.5% for dust, 17% for cotton, 15.8% for tobacco, 14.8% for fungi and 12.5% for wood. Allergic respiratory symptoms in at least one parent were reported in 20.5% of the sample.

Table 2 shows the statistical associations between exposure to passive smoking and the presence of other variables studied. We found a statistically significant association between parents who had atopic disorders and children not exposed to passive smoking.

**Table 2:** Association between exposure to passive smoking in children and other variables

Passive smoking(%)			
	Not Exposed	Exposed	^p^
Damp, musty house			
Yes (n = 30)	11.3	6.5	0.494
No (n = 138)	46.4	35.7	
Pet			
Yes (n = 65)	23.6	15.8	0.611
No (n = 100)	33.9	26.7	
Breastfeeding			
Yes (n = 155)	54.2	38.1	0.379
No (n = 13)	3.6	4.2	
Positive skin tests for allergies (2 or more)			
Yes (n = 23)	16.0	12.3	0.645
No (n = 58)	44.4	27.2	
Parents with atopic disorders			
Yes (n = 53)	24.8	10.7	0.036
No (n = 106)	33.6	30.9	
Full time at school			
Yes (n = 118)	41.3	34.8	0.397
No (n = 37)	14.8	9.0	

Table 3 draws attention to the presence or absence of respiratory problems and asthma attacks during the preceding three months in the studied sample, in relation to unhealthy dwelling places, presence of a pet at home, breastfeeding, presence of two or more positive tests for inhaled antigens, atopic disorders in parents, smoking parents, and the fact that the children attended school full-time. Statistical significance was demonstrated for the associations of atopic disorders in parents with asthma attacks and higher incidence of respiratory infections during the preceding three months.

**Table 3:** Odds ratio (and 95% confidence interval) for the association between presence of either respiratory infections or asthma attacks over the preceding three months and environmental variables

	Respiratory infections	Asthma
Damp, musty house	0.91 (0.43-1.93)	1.59 (0.71-3.57)
Pet	1.20 (0.66-2.20)	0.54 (0.26-1.13)
Breastfeeding	0.81 (0.27-2.40)	0.76 (0.23-2.46)
Positive skin tests for allergies (2 or more)	1.06 (0.41-2.72)	0.37 (0.11-1.33)
Parents with atopic disorders	0.40 (0.20-0.79)	3.23 (1.59-6.6)
Smoking parents	0.87 (0.47-1.61)	1.20 (0.59-2.41)
Full time at school	0.88 (0.43-1.84)	1.69 (0.66-4.31)

Parents reported that 51% of the children had had one or more respiratory infections during the preceding 3 months (including pneumonia, rhinitis, otitis media, sinusitis or pharyngitis). They also reported that 25.7% of the children had asthma and 52.1% of the sample had had one or more asthma attacks during the preceding three months.

**Figure 1:**
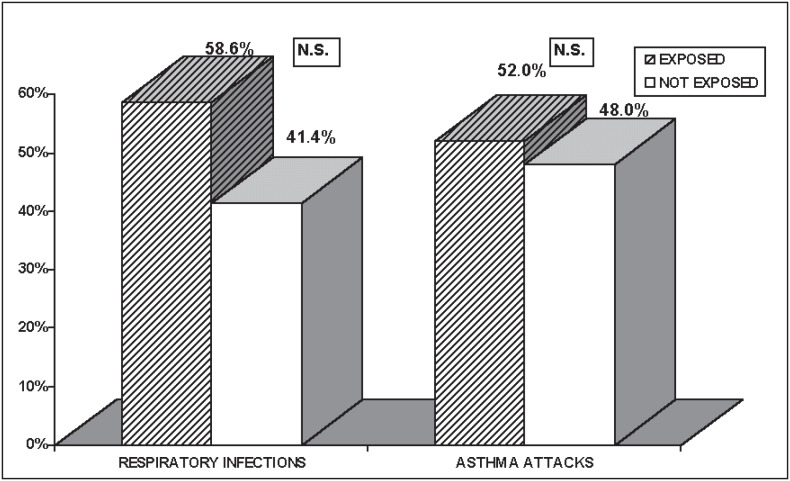
Association between respiratory infections and asthma attacks among children over the preceding 3 months, according to exposure to environmental tobacco smoke.

Figure 1 shows the respiratory infections and asthma attacks in children exposed to tobacco smoke in comparison with those not exposed. However, no statistical significance could be found.

## DISCUSSION

There was quite a high prevalence of children with parents who were active smokers (37.8%), and the mean cigarette use (10-19 cigarettes/day) was similar to that of the Brazilian population.^11^

With regard to the characteristics of their homes, most families had good housing conditions (less than 28% of the households were considered damp and/or musty). Moreover, a large number of children shared their bedrooms with only one more person, which decreases exposure to respiratory infections. Their socioeconomic status was also considered satisfactory in comparison with the Brazilian population. In the general population, only 50% belong to the B and C classes,^10^ whereas we noted that among our studied population 86% belonged to these social classes. This may have decreased the risk of acquiring respiratory infections, even when exposed to tobacco smoke.^5,2^

The positive history of breastfeeding was quite satisfactory (92.3%), since it is known that, for offspring who have been breastfed, the occurrence of respiratory infections is reduced, although this is more relevant in early childhood.^12^ Nationwide data has shown an increasing number of breastfed infants over the last few years. In the southeastern region of Brazil, 58% (47-67%) of the newborn are breastfed for at least 4 months.^12^ Breastfeeding probably causes a protective biological effect against the irritation from tobacco smoke in the children's respiratory tract. Among the infants that were breastfed for more than 6 months, there was no increase in the risk of lower respiratory tract illnesses caused by the mother's smoking.^13^

Statistical significance was observed in the association between atopic disorders in parents of children with asthma attacks and the presence of respiratory infections during the preceding 3 months, but no association between 2 or more positive skin tests for allergies and asthma could been shown. It is known that both asthma and a greater vulnerability to infections are directly related to atopic disorders and, a familiar genetic component that is next in this causal link.

Today it is known that the offspring of smoking mothers have a higher probability of developing asthma, independent of other factors.^7^ It has been demonstrated that the addition of two other factors, such as atopic disorders and passive exposure to tobacco smoke, greatly increases the risk of triggering respiratory infections in children whose mothers smoke,^6^ especially for infants in their first year of life.^14-16^ A higher incidence of respiratory symptoms among children aged 0 to 10 years who were exposed to passive smoking at home was demonstrated by Botelho et al.^17^.

We found that parents with atopic disorders tried to preserve their kids from passive smoking and they exhibited reduced occurrence of respiratory infections (OR = 0.40), even though the children had had higher episodes of asthma attacks (OR = 3.23) over the preceding three months (Table 2 and 3).

In this research, we did not find an increase in the incidence of respiratory infections and asthma attacks among children exposed to tobacco smoke. This can be explained by the fact that we were dealing with older children with a good socioeconomic status and satisfactory housing, who had been adequately breastfed. The fact that they were away from home for longer periods (76.1% were at school all day long) reduced their exposure to environmental tobacco smoke at home. Moreover, the population studied was small and the time period over which the incidence of respiratory infections and asthma attacks was investigated was limited to only three months, because the longer the period that the parents are asked about, the lower the fidelity of data is. In addition to this, the study was performed in a very polluted city (São Paulo), and this may have contributed to some confusion in the documentation of higher occurrence of respiratory infections and asthma attacks related to passive smoking exposure. Such problems could be clarified with a study conducted using a larger and more representative sample of the population, observed over a prolonged period of time.

## CONCLUSIONS

The presence of respiratory infections and asthma attacks were associated with atopic parents but not with the presence of two or more positive skin tests for allergies among the children studied. Also, the presence of respiratory infections and asthma were not associated with parents who smoke, or with studying full-time at school.

## References

[1] Chilmoncyk BA, Knight GJ, Pulomaki GE (1990). Environmental tobacco smoke during infancy. Am J Public Health.

[2] Harlap S, Davies AM (1974). Infant admissions to hospital and maternal smoking. Lancet.

[3] Ferguson DM, Horwood LJ, Shannon FT (1981). Parental smoking and lower respiratory illness in the first three years of life. J Epidemiol Commun Health.

[4] Bonham GS, Wilson RA (1981). Children's health in families with cigarette smokers. Am J Public Health.

[5] Colley JRT, Holland WW, Corkhill RT (1974). Influence of passive smoking and parental phlegm on pneumonia and bronchitis in early childhood. Lancet.

[6] Kraemer MJ, Richardson MA, Weiss NS (1983). Risk factor for persistent middle-ear effusions, otitis media, catarrh, cigarette smoke exposure, and atopy. JAMA.

[7] Gortmaker AS, Walker DK, Jacobs FH (1982). Parental smoking and the risk of childhood asthma. Am J Public Health.

[8] Berkey CS, Ware JH, Dockery DW (1986). Indoor air pollution and pulmonary function growth in pre-adolescent children. Am J Epidemiol.

[9] Taskin DP, Clark VA, Simmons M (1984). The UCLA population studies of chronic obstructive pulmonary disease. VII. Relationship between parental smoking and children's lung function. Am Rev Respir Dis.

[10] Sociedade Brasileira de Pesquisa de Mercado (1997). Critério de Classificação Econômica Brasil.

[11] (1988). Ministério da Saúde: Pesquisa Nacional sobre Estilo de Vida.

[12] Venancio SI, Monteiro CA (1998). A tendência da prática da amamentação no Brasil nas décadas de 70 e 80. Rev Bras Epidemiol..

[13] Galve R, Garcia VC, Rubio SFJ, Penascal PE, Jimenez HJM, Martinez BJA (1998). Passive smoking and other risk factors associated to the lower respiratory illnesses in infants. Aten. Primaria.

[14] Rosemberg J (1990). Tabagismo e doenças respiratórias. JBM.

[15] Rosemberg J (1982). Poluição ambiental tabágica. JBM.

[16] Rosemberg J (1985). Nocividade à saúde das crianças, conseqüente do tabagismo dos pais. Rev Ass Med Bras.

[17] Botelho C, Barbosa LSG, Silva MD, Barros MD (1987). Sintomas respiratórios e tabagismo passivo em crianças. J Pneumol.

